# Case Report: Reversal and subsequent return of optic disc cupping in a myocilin (MYOC) gene-associated severe Juvenile Open-Angle Glaucoma (JOAG) patient

**DOI:** 10.12688/f1000research.127871.1

**Published:** 2022-11-22

**Authors:** Hani El Helwe, Sandy Samuel, Sanchay Gupta, Cameron Neeson, Marika Chachanidze, David A. Solá-Del Valle

**Affiliations:** 1 Glaucoma Service, Massachusetts Eye and Ear Infirmary, Boston, Massachusetts, 02114, USA; 2 Massachusetts Eye and Ear, Harvard Medical School, Boston, Massachusetts, 02115, USA

**Keywords:** Reversal of optic nerve head cupping, Juvenile open-angle glaucoma, Myocilin gene, glaucoma filtration surgery

## Abstract

To our knowledge, this case report describes the first instance of reversal of glaucomatous optic nerve cupping in a young adult with a rare form of juvenile open-angle glaucoma (JOAG) associated with a novel variant of the myocilin gene (MYOC). This 25-year-old woman with severe-stage MYOC-associated JOAG presented with blurry vision and intermittent pain in her left eye. She had a strong family history of glaucoma in multiple first-degree relatives with an identified novel variant of MYOC. Examination revealed intraocular pressures (IOPs) of 10 mmHg OD and 46 mmHg OS, with cup-to-disc ratios of 0.90 and 0.80. The patient experienced substantial reversal of optic disc cupping OS following dramatic IOP reduction with trabeculectomy, and subsequently experienced a return of cupping after an IOP spike 15 months postoperatively. The reversal of cupping did not correspond to any changes in the patient’s visual field. After an initial decrease in retinal nerve fiber layer (RNFL) thickness, RNFL remained stable for over 2 years after trabeculectomy as seen on Optical Coherence Tomography (OCT). This case suggests reversal of cupping can occur well into adulthood in a MYOC-associated JOAG patient, and it demonstrates the potential bidirectionality of this phenomenon. Moreover, it suggests that these structural changes may not correspond to any functional changes in visual fields or RNFL thickness.

## Introduction

The myocilin gene (MYOC) is located within the glaucoma locus GLC1A present on chromosome 1q21-q31. ^1^ It encodes an extracellularly-secreted matrix protein expressed by many tissues throughout the body but only found to cause disease at the level of the trabecular meshwork (TM). ^2^ MYOC mutations have been implicated in 8-36% of cases of familial juvenile open-angle glaucoma (JOAG) which is characterized by very high intraocular pressures (IOPs), optic disc cupping, and visual field loss at a young age (<40 years). ^3^^,^^4^ The exact mechanism by which MYOC exerts its function isn’t fully understood, but it is believed to be intimately involved in dictating TM stiffness and resistance to flow. ^5^^,^^6^ There are currently 280 identified MYOC mutations, 37.86% of which have been associated with the development of glaucoma. ^6^^–^^8^ These mutations are thought to cause aqueous outflow obstruction at the level of the TM. Missense mutations make up 85.9% of glaucoma-related MYOC mutations, most of which arise within the olfactomedin (OLF) domain present on exon 3 of MYOC. ^9^ One such mutation carried by the patient presented in this report is Glu385Lys. This mutation was initially discovered in her sibling and was later found to be present in all her family members affected by glaucoma and absent in those unaffected by the disease. Glu385Lys exchanges a highly-conserved negatively-charged glutamic acid for a positively-charged lysine amino acid. ^7^ This substitution causes a conformational change at the level of the OLF domain, which disrupts proper processing and delivery of the MYOC protein to the extracellular matrix. Studies hypothesize that misfolded MYOC proteins are then retained within the endoplasmic reticulum (ER) of TM cells. ^10^^–^^14^ These retained MYOC proteins trigger a stress response within the ER that eventually leads to the apoptosis of the TM cells. ^12^^,^^15^^,^^16^ These morphological changes impact TM biomechanics causing increased stiffness and reduced drainage capability, which give rise to JOAG.

Previously described in other JOAG patients, reversal of optic disc cupping refers to the improvement in the appearance of the optic nerve head in response to a marked decrease in IOP, often following surgical intervention. ^17^ While this structural phenomenon is widely recognized as an indicator of successful treatment in pediatric glaucoma patients, it is less commonly described in adults. ^18^^,^^19^ Moreover, there is controversy as to whether these structural improvements correspond to any functional improvements in patients’ visual fields and optical coherence tomography (OCT). ^20^^,^^21^

This case report describes reversal of optic disc cupping in a 25-year-old woman with severe-stage MYOC-associated JOAG, and eventual return-of-cupping following a period of less tightly controlled IOP. Additionally, using fundus photos, Humphrey Visual Field (HVF), and OCTs, we suggest these structural changes did not appear to correspond to functional disease changes.

To the best of our knowledge, this is the first time this phenomenon has been described in a MYOC-associated JOAG patient.

## Case presentation

The case involves a 25-year-old Puerto Rican woman with severe-stage MYOC-associated JOAG (first diagnosed at age 20) who presented to the Massachusetts Eye and Ear Glaucoma Clinic in February 2018 with blurry vision and intermittent pain in the left eye (OS). She had a strong family history of glaucoma in multiple first-degree relatives, and she was found to share a novel variant of the MYOC gene (NM_000261.2: c.1153G>A, p.Glu385), as described above, with her father, sister, and brother under a research protocol completed at the Yale Center for Genome Analysis. ^7^ The patient had previously undergone successful trabeculectomy with mitomycin C in the right eye (OD) in 2014 and selective laser trabeculoplasty (SLT) in the left eye in early 2018 that failed to significantly lower her IOP. Her maximum recorded IOP was 40 mmHg OD and 46 mmHg OS.

On examination in February 2018, the patient’s best corrected visual acuity (BCVA) was 20/25 OD and 20/30 OS. Her IOP was 10 mmHg OD off glaucoma medications and 46 mmHg OS on dorzolamide twice a day (bid), timolol bid, brimonidine bid, and bimatoprost at night (qhs).

**Figure 1.**
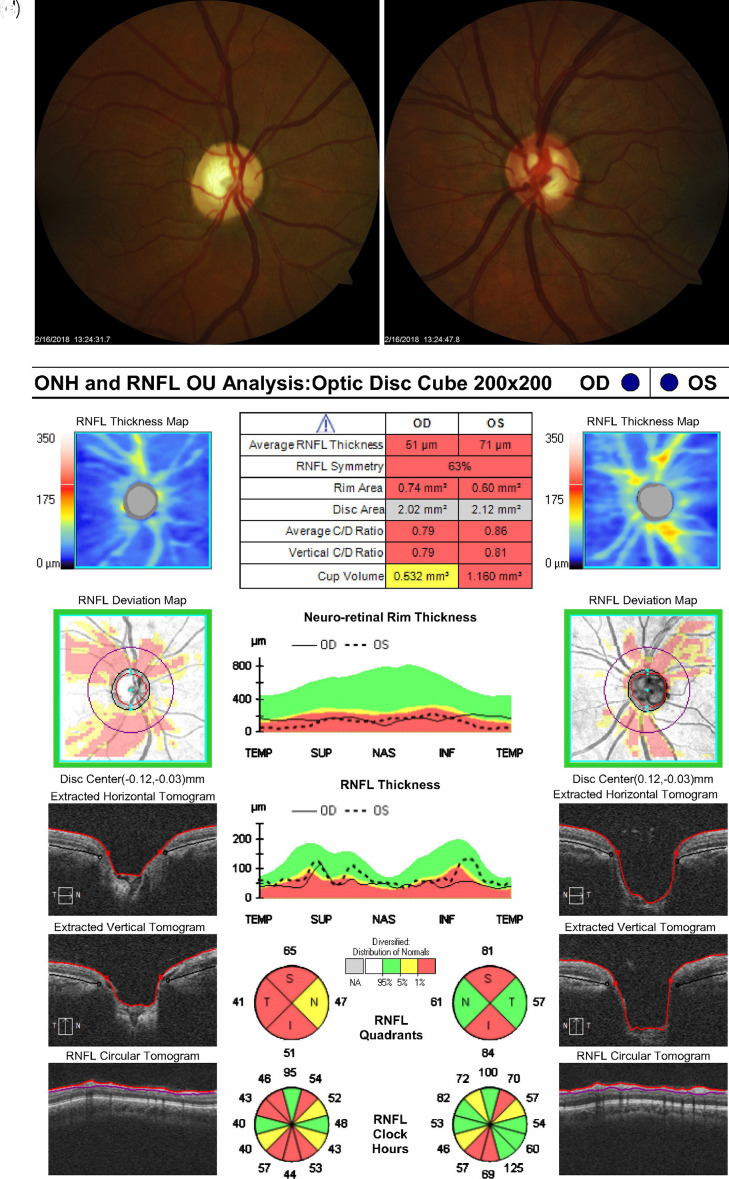

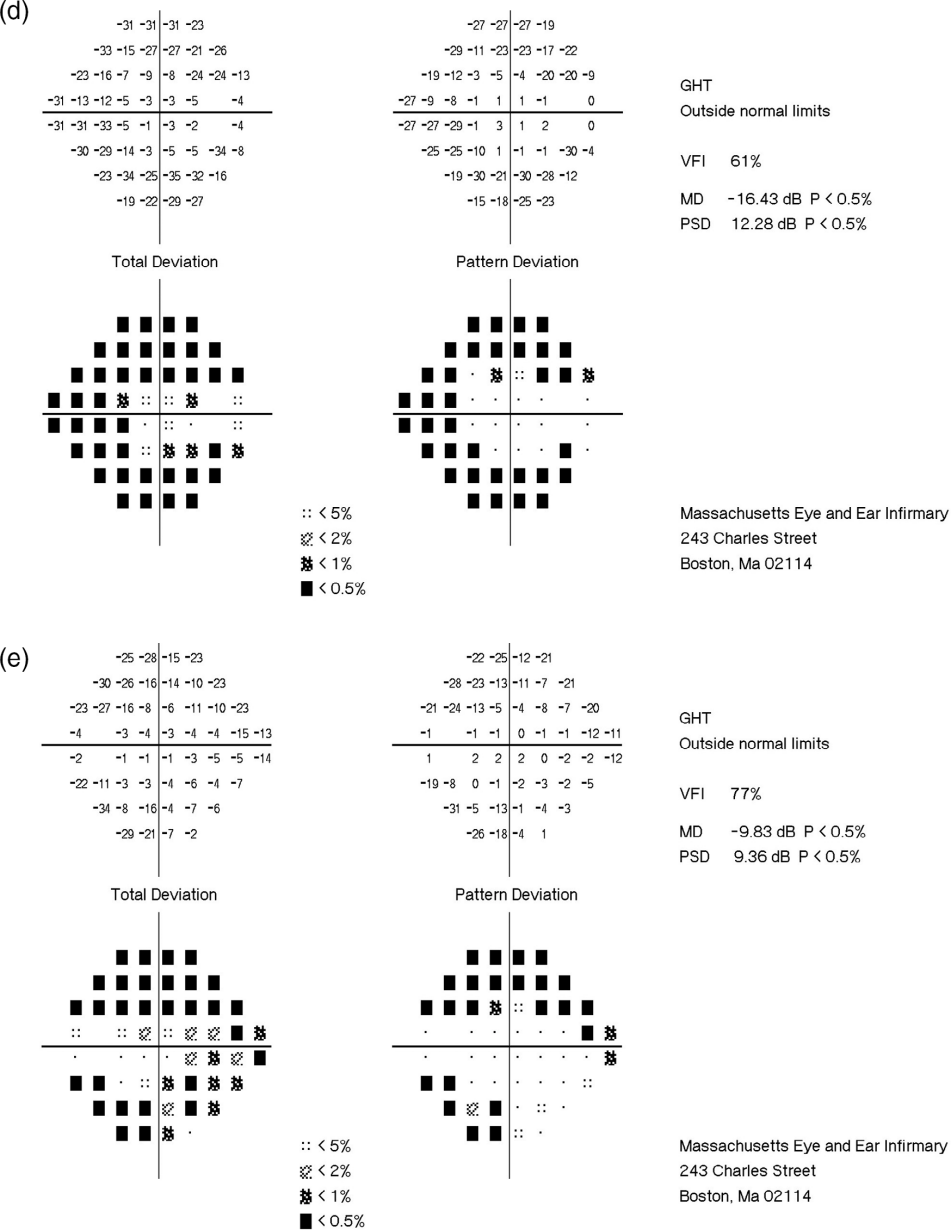
Optic nerve head photo, Humphrey Visual Field (HVF), and Optical Coherence Tomography (OCT) in February 2018 for both eyes. a. Optic nerve head OD (2/16/2018). b. Optic nerve head OS (2/16/2018). c. OCT ONH and RNFL Analysis OU (2/13/2018). d. HVF OD (2/13/2018). e. HVF OS (2/13/2018).

Examination of both optic nerves revealed diffuse thinning of the disc without hemorrhage bilaterally with a cup-to-disc ratio of approximately 0.90 OD and 0.80 OS. HVF analysis revealed constriction with central island OD and superior arcuate more than inferior arcuate OS. OCT revealed diffuse thinning of the retinal nerve fiber layer (RNFL) OU (shown in Figure 1).

Given the extremely elevated IOP in her left eye despite receiving close to maximally tolerated medical therapy (MTMTx), the patient underwent an urgent trabeculectomy with mitomycin-C (concentration of 0.4 mg/mL) in February 2018. The patient’s IOP and BCVA OD have remained stable since her initial presentation, ranging from 10-12 mmHg and 20/20 to 20/25, respectively. Subsequent discussion is therefore focused primarily on her left eye with relevant visit data for the left eye provided in Table 1. On postoperative day one, her IOP was 05 mmHg OS off glaucoma medications. Her slit lamp and fundus exam revealed a deep anterior chamber, cornea without Descemet’s folds, and a flat macula. The anterior chamber showed 1+ cell. Three days following her procedure, a leak was seen along the limbus and a bandage contact lens (BCL) was placed in the office. Anterior chamber inflammation resolved by her one-week postoperative follow-up. At this time the BCL was removed, and the leak along the limbus became evident again. Due to persistent leakage along the conjunctiva superiorly the patient underwent a revision where 8-0 Vicryl mattress sutures were used to repair the leak. One day after the first revision, a slow leak persisted along the superonasal aspect of the peritomy, and the patient’s IOP dropped to 02 mmHg. This was the only instance when IOP was in the hypotonous range, although a deep central anterior chamber, absence of corneal Descemet’s folds, and a flat macula were noted. This necessitated a second bleb revision in March 2018 with more 8-0 Vicryl and several 10-0 Nylon mattress sutures. Her IOP was subsequently noted to be 06 mmHg with no signs of hypotony or leak.

**Table 1.** Summary of patient visit data OS from February 2018 to January 2022.

Figure	Visit type	Visit date	IOP (mmHg)	BCVA	RNFL (μm)	HVF Median Deviation (dB)	HVF Median Deviation Δ from baseline	C/D ratio
**1**	Preoperative; OCT ONH and RNFL; HVF	2/13/2018	46	20/30	71	-9.83	baseline	0.8
	POD1, trabeculectomy w/MMC	2/21/2018	05	20/70				0.8
	POD1, revision 1	3/3/2018	02	20/40				0.4
	POD1, revision 2	3/7/2018	06	20/50				0.4
**2**	Follow-up; OCT Macula	4/3/2018	06	20/30				0.35
**3**	Follow-up; OCT ONH and RNFL	10/11/2018	05	20/40	56			0.45
**4**	Follow-up	5/23/2019	23	20/25				0.70
**5**	Follow-up; OCT ONH and RNFL; HVF	11/14/2019	09	20/40	52	-9.38	+0.45	0.70
	Follow-up; HVF	2/20/2020	09	20/30		-11.15	-1.32	0.70
**6**	Follow-up; OCT ONH and RNFL	6/23/2020	09	20/40	53			0.7
	Follow-up; HVF	10/20/2020	11	20/40		-10.62	-0.79	0.70
	Follow-up; OCT ONH and RNFL	2/16/2021	09	20/40	54			0.7
	Follow-up; HVF	6/29/2021	09	20/25		-9.40	+0.43	0.7
	Follow-up; OCT ONH and RNFL	1/13/2022	07	20/30	58			0.7

**Figure 2.**
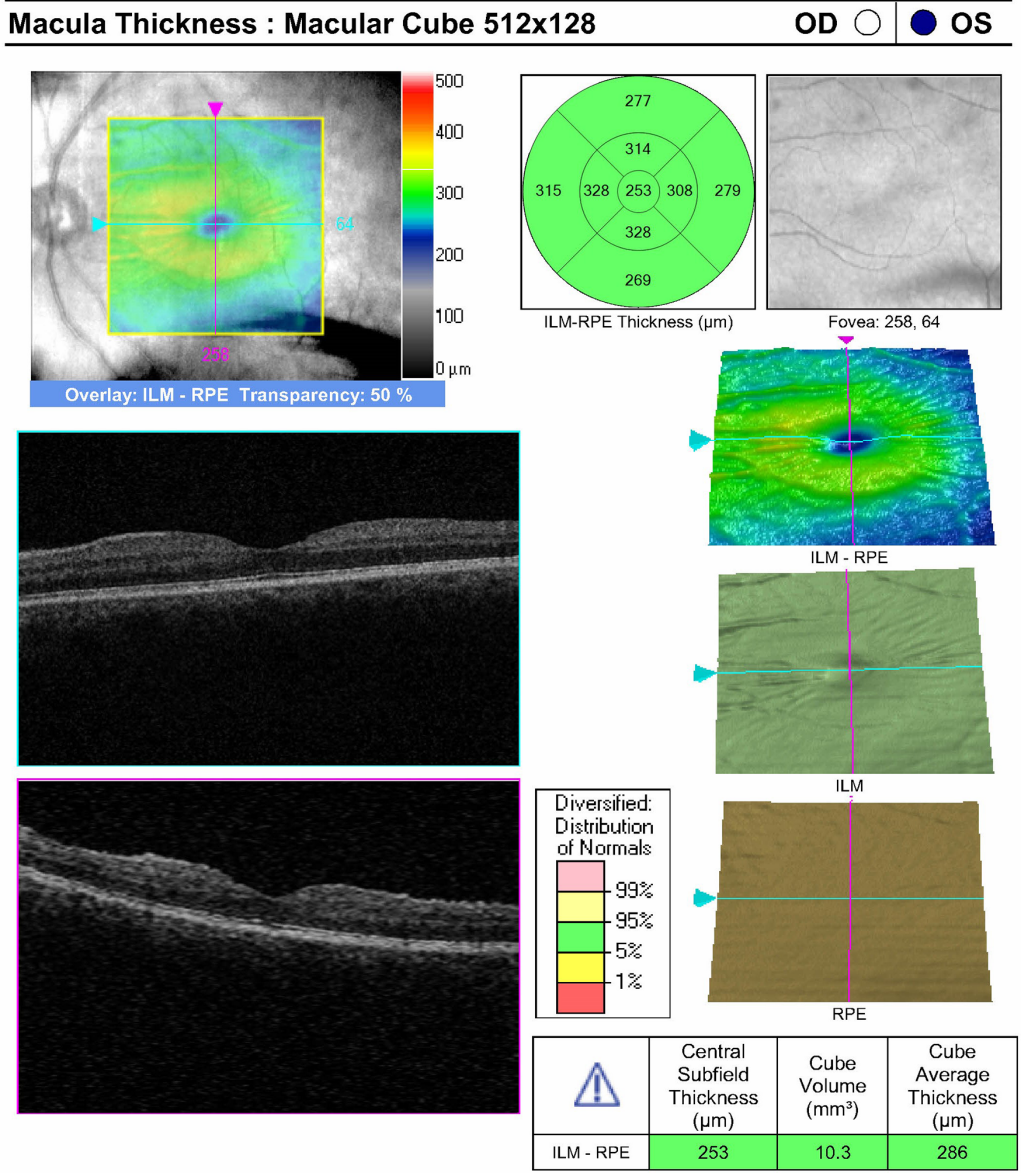
Optical Coherence Tomography (OCT) OS for Macula Thickness in April 2018 (4/3/2018).

**Figure 3.**
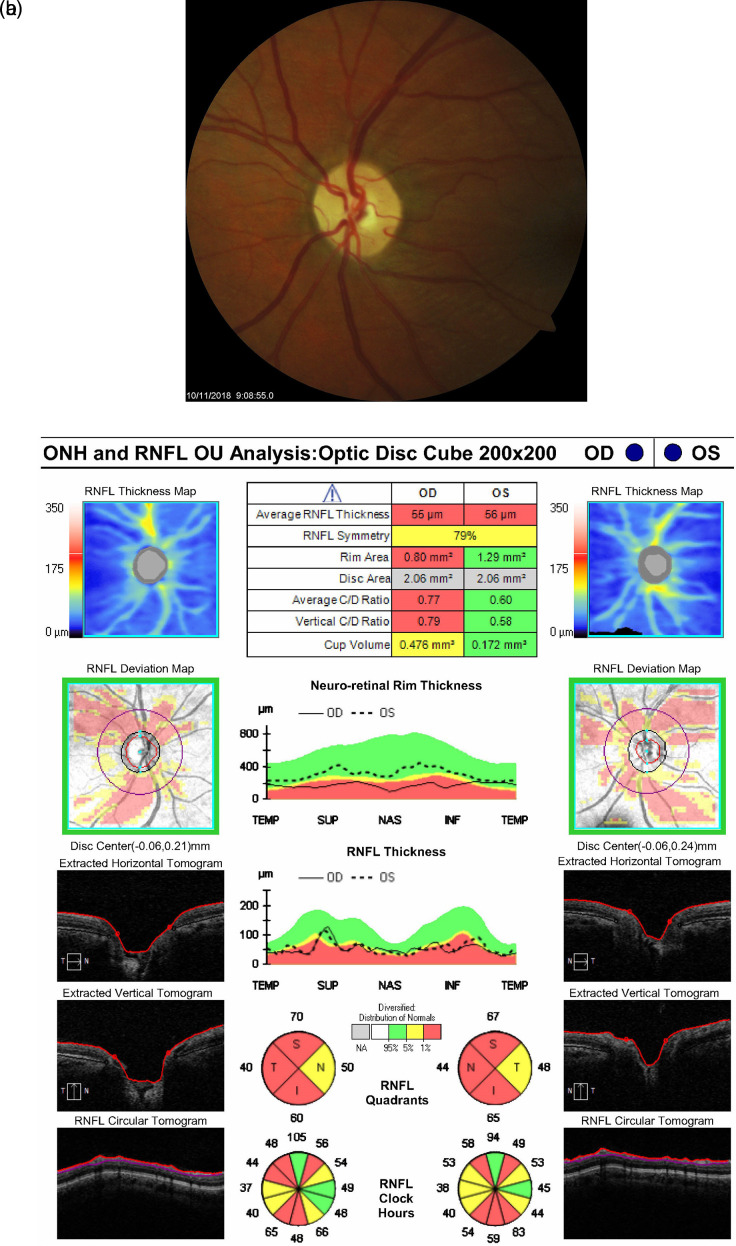
Optic nerve head photo OS and Optical Coherence Tomography (OCT) in October 2018 for both eyes. a. Optic nerve head OS (10/11/2018). b. OCT ONH and RNFL Analysis OU (10/11/2018).

**Figure 4.**
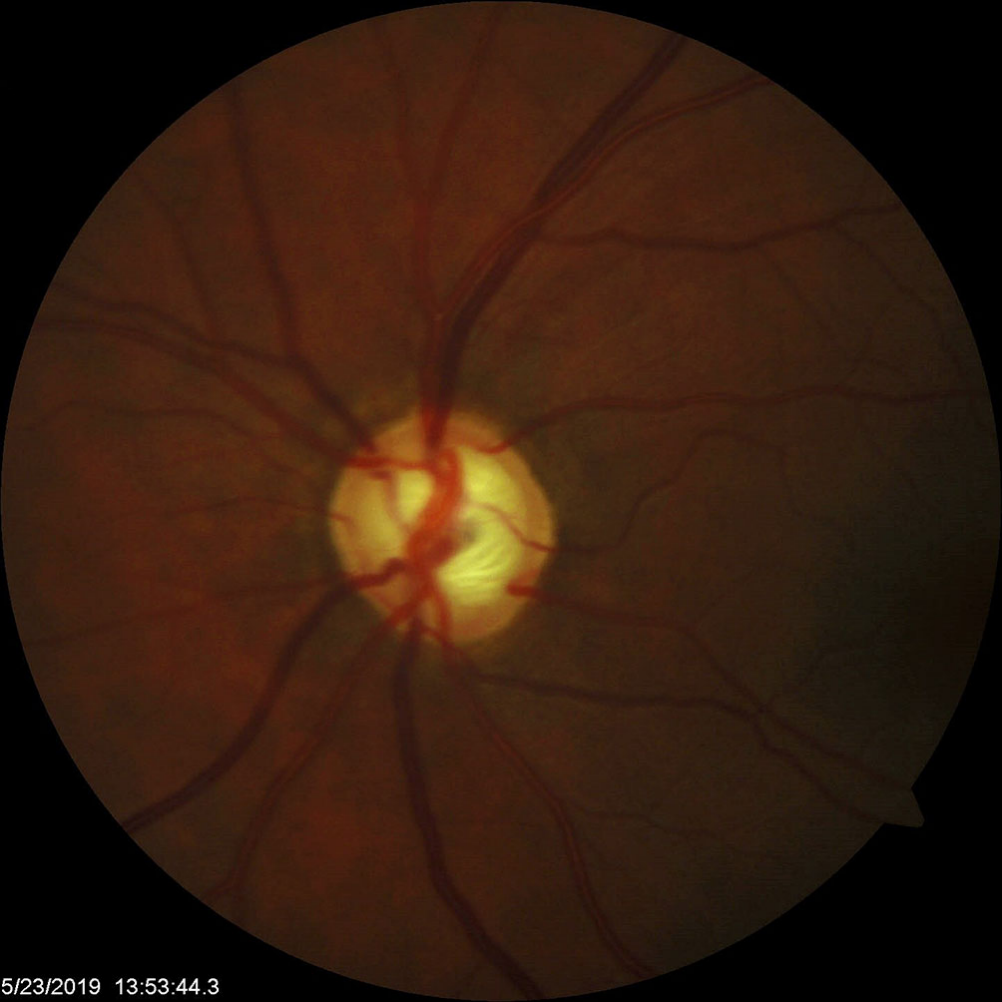
Optic nerve head photo OS in May 2019 OS (5/23/2019) demonstrating return of optic nerve cupping.

**Figure 5.**
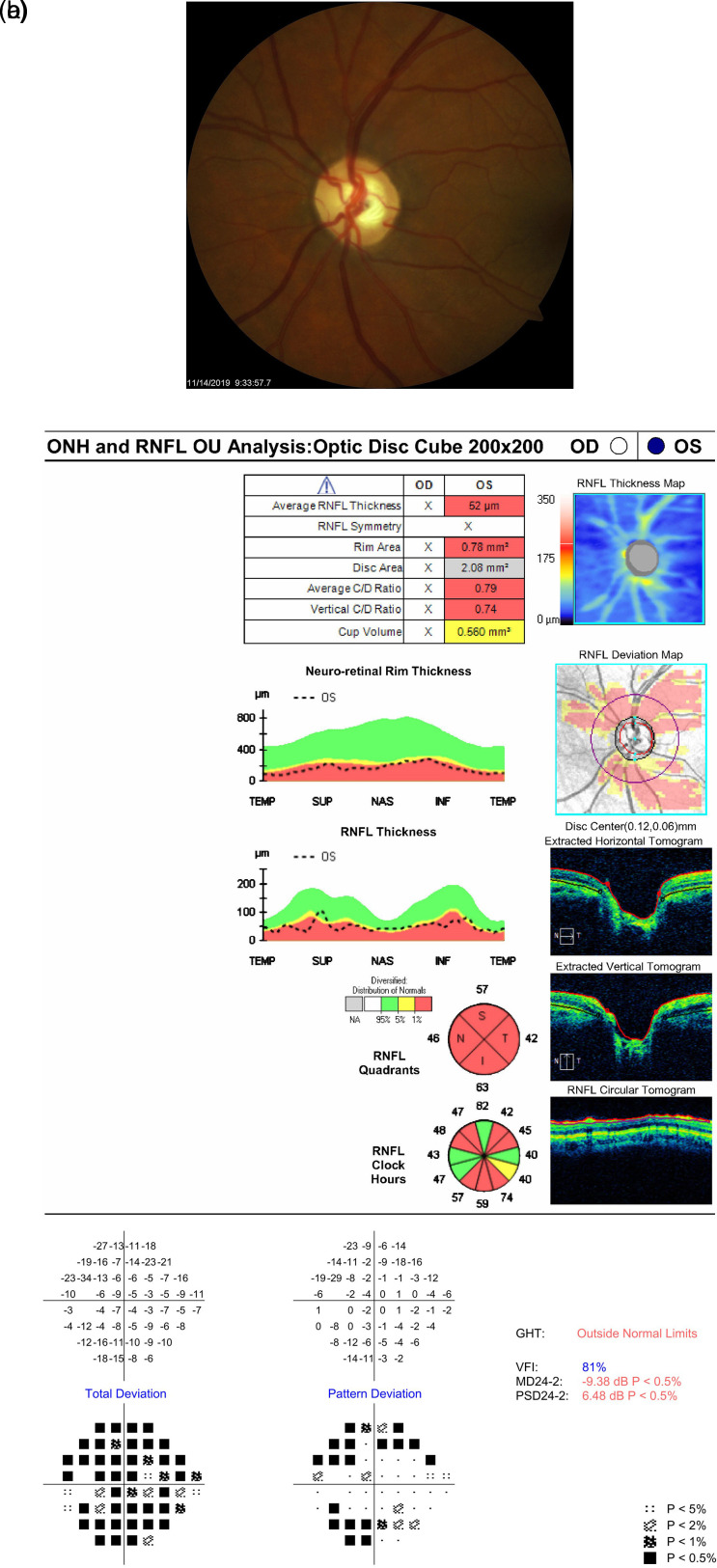
Optic nerve head photo, Humphrey Visual Field (HVF), and Optical Coherence Tomography (OCT) in November 2019. a. Optic nerve head OS (11/14/2019). b. OCT ONH and RNFL Analysis OU (11/14/2019). c. HVF OS (11/14/2019).

**Figure 6.**
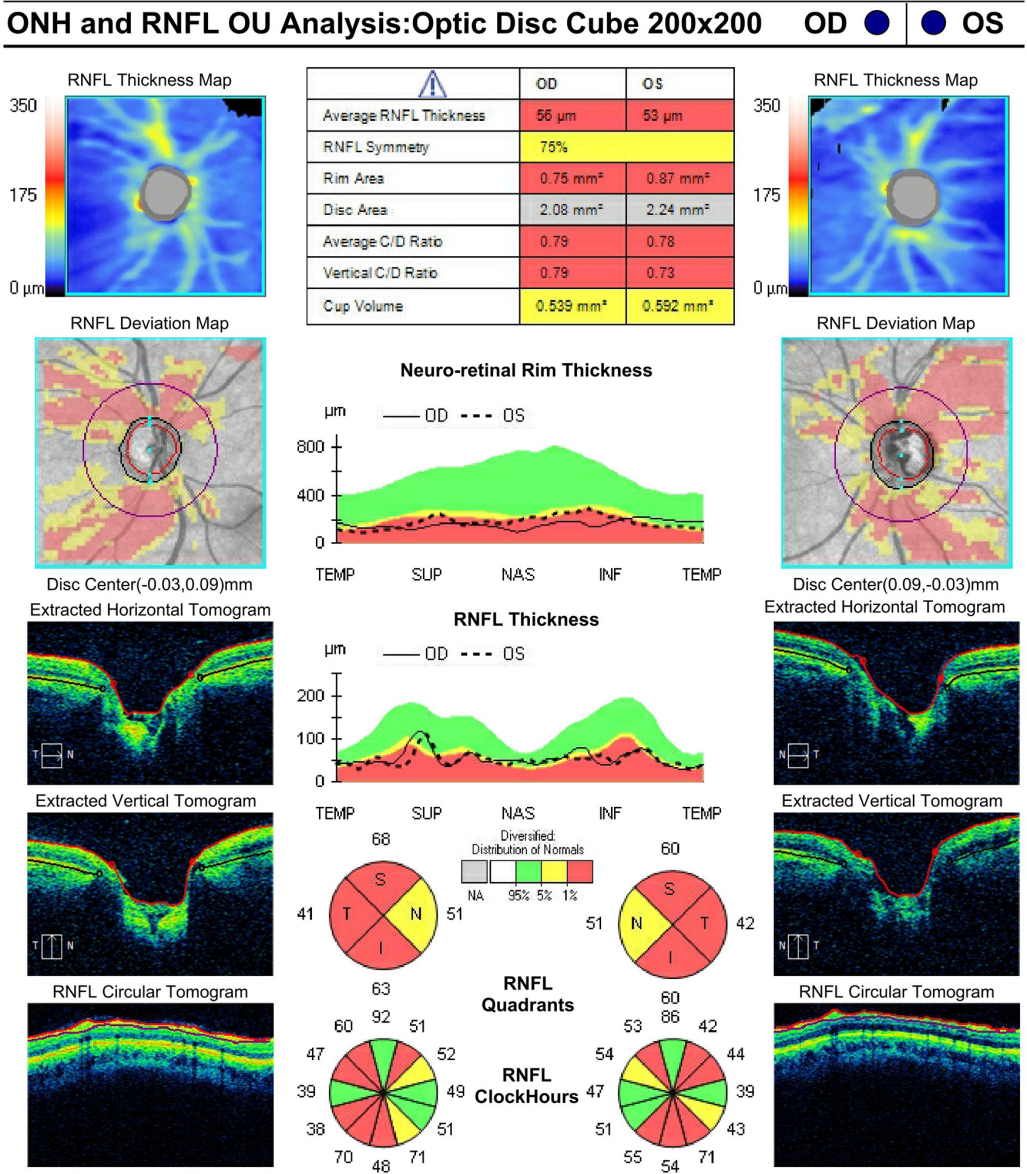
Optical Coherence Tomography (OCT) ONH and RNFL Analysis for both eyes in June 2020 (6/23/2020).

OS = left eye; IOP = intraocular pressure; OCT = optical coherence tomography; ONH = optic nerve head; RNFL = retinal nerve fiber layer; HVF = Humphrey visual field; POD = postoperative day; BCVA = best corrected visual acuity; Δ = change; C/D = cup-to-disc; 1,2,3,4,5,6 = Figures 1, 2, 3, 4, 5, 6.

In April 2018, a reliable macula OCT revealed normal values for central subfield thickness, cube volume, and cube average thickness, indicating absence of macular folding or signs of macular hypotony even when the cup-to-disc ratio was noted to be 0.35 (shown in Figure 2). Eight months after the initial surgery her BCVA was 20/25 OD and 20/40 OS, and IOP measurements were 10 mmHg OD and 05 mmHg OS off glaucoma medications. Her exam continued to show no signs of hypotony. Photographs of the left optic disc eight-months postoperatively demonstrated substantial reversal of cupping with a reduced cup-to-disc ratio of 0.45 (shown in Figure 3). Although the patient’s first postoperative OCT showed a decrease in RNFL thickness from 71 μm OS preoperatively to 56 μm OS, this thickness remained stable throughout her follow-ups. It is also worth noting that the patient’s average neuro-retinal rim thickness was higher at the October 2018 visit compared to preoperative measurements, further corroborating reversal of cupping of the nerve head (shown in Figure 3).

In May 2019, the patient’s IOP OS spiked to 23 mmHg and her cup-to-disc ratio increased to 0.70, indicating a return to cupping of the nerve. After the IOP spike, the patient was placed on timolol OS, and her IOP has been controlled at subsequent follow-ups. It is worth noting that the RNFL and HVF remained stable after the nerve became cupped again in May 2019 (shown in Figure 4). From her November 2019 follow-up to her most recent follow-up in January 2022, her cup-to-disc ratio has remained steady at 0.70 throughout all visits. Additionally, her average neuro-retinal rim thickness returned to below normal levels and has remained there, as seen in Figures 5 and 6. In contrast, her RNFL remained stable at 53 μm compared to 56 μm in October 2018 (shown in Figure 6). Additionally, her HVF has remained stable throughout the preoperative and all postoperative follow-ups, with a maximum deviation of -1.32 dB from baseline. These fluctuations are within the expected test-retest variability range and will be explained in the discussion. For the patient’s follow-ups after November 2019, her IOP has been stable, ranging from 09-11 mmHg OS. At her latest follow-up in January 2022, the patient’s BCVA was 20/30 OS, with an IOP of 07 mmHg OS. The patient is currently on timolol bid OS.

## Discussion

The current case describes the reversal of optic disc cupping after substantial IOP reduction in an adult patient with severe-stage MYOC-associated JOAG. We also describe a significant return towards the patient’s preoperative cup-to-disc ratio following an IOP spike. The patient’s cup-to-disc ratio was noted to be 0.70 in their May 2019 follow-up and has remained consistent for over two years. Corresponding HVF and OCT analyses do not appear to show significant corresponding functional changes. Reversal of cupping has been described in only a select few adult patients following substantial reductions in IOP. Specifically, Pederson *et al.* reported five adult cases (31-62 years of age) where reversal of cupping was observed after an IOP reduction of 68% following filtration surgery. ^19^ Similar to this case, one of these patients experienced an IOP spike 10 months later that led to a return of cupping to preoperative size. In addition, Esfandiari *et al.* showed a positive correlation between the degree of cupping reversal and the magnitude of IOP reduction (regression coefficient = 0.251, P = 0.02). ^22^ Interestingly, the authors found a negative correlation between the extent of cupping reversal and age (regression coefficient = -0.224, P = 0.04) as well as cup-to-disc ratio (regression coefficient = -0.212, P = 0.05). In short, a larger IOP reduction and younger age were correlated with larger reversals of cupping and degrees of functional improvement in the HVF.

Some authors suggest that macular hypotony after glaucoma surgery may trigger ocular changes that mediate optic nerve cupping reversal in such cases. ^23^^,^^24^ However, exam and imaging findings from our patient’s postoperative follow-up visits suggest that macular hypotony is not a significant driver for her cupping reversal as her IOP was below 05 mmHg only once for a day without ocular signs of hypotony. Her exams were notable for a deep central anterior chamber and absence of significant Descemet membrane folds while macular OCT demonstrated normal central subfield thickness with no evidence of choroidal or retinal folding (shown in Figure 2).

In the current case, we did not observe any functional changes on HVF or OCT that would correspond to the structural changes observed at the optic nerve head. Horani *et al.* and Tan *et al.* quantified the test-retest variability of HVF and OCT imaging, reporting mean variability among repeat tests of 2.44 dB on HVF and 4.89 μm on Cirrus OCT. ^25^^,^^26^ Table 1 shows stability in the patient’s HVF over all follow-ups with median deviations from baseline falling within the expected ±2.44 dB range. Although RNFL thickness decreased after the initial surgery from 71 μm OS to 56 μm OS in October 2018, BCVA and HVF were virtually unchanged over this interval and average RNFL thickness has remained stable in the mid-50s for over two years in subsequent reliable OCTs. Interestingly, Kim *et al.* found that patients with a high preoperative peak IOP (≥37 mmHg) exhibited significant average RNFL thinning after IOP lowering surgery compared to their preoperative average RNFL thickness. ^27^ We hypothesize that the higher preoperative RNFL value in this case may have been artificially elevated in the setting of extremely high IOP (46 mmHg) and appears to subsequently stabilize after surgery. Furthermore, when the patient experienced cupping reversal, her average neuro-retinal rim thickness increased from preoperative baseline (compare Figures 1 and 3). The neuro-retinal rim is the area of the optic disc occupied by the retinal nerve fiber axons. Analyzing its changes in pattern of thickness and thinning are important for detecting the extent of disc damage. When the patient’s optic nerve cupped again, her average neuro-retinal rim thickness returned to below normal levels and has remained there in subsequent follow-ups, consistent with nerve cupping (shown in Figures 5 and 6).

In contrast, Leung *et al.* reported reversal of cupping in a 20-year-old woman with corresponding functional improvements in RNFL thickness. ^28^ However, the duration of these structural and functional improvements was not reported. Notably, adult cases of cupping reversal are limited to patients in the early stages of glaucoma, unlike the current severe-stage patient. For instance, all the patients reviewed by Pederson *et al.* were in the early stages of glaucoma and Leung *et al.* described a case of mild JOAG. ^19^^,^^28^ None of these patients were known to have glaucoma associated with an identified genetic mutation.

To the best of our knowledge, this is the only case to report reversal of cupping in an adult patient with MYOC-associated severe JOAG. While we are unable to infer a causal link between the MYOC variant and this patient’s cupping reversal after trabeculectomy, this case may shed light on the structural and functional consequences of IOP changes in patients with this genetic mutation.

To conclude, this case reflects the responsiveness of the optic nerve head to IOP-lowering surgery in a patient with a rare form of MYOC-associated severe JOAG. Furthermore, it describes a return of optic nerve cupping corresponding to a resurgence in IOP. This highlights the bidirectional quality of this phenomenon, and the relatively short timescale in which cupping can resume after a period of suboptimal IOP control. Moreover, HVF and OCT testing did not seem to reveal a corresponding functional change in the patient’s disease, suggesting that such a structural change may not indicate any significant functional disease changes in MYOC-associated JOAG.

### Statement of ethics

Ethical approval was not required for this study in accordance with local or national guidelines. Written informed consent was obtained from the patient for publication of the details of their medical case and any accompanying images. A copy of the written consent is available for review by the Editor of the journal.

## Author contributions

Dr. Hani El Helwe, Sandy Samuel, and Sanchay Gupta contributed equally to this work as co-first authors. These authors substantially acquired, analyzed, and interpreted the data for this work. Additionally, they drafted and revised the work to be as up to date as possible. They have read and approved of the final version to be published and agreed to be accountable for all aspects of the work. Dr. Marika Chachanidze, and Cameron E. Neeson initiated this case report, performing the initial data acquisition, analysis, and manuscript drafting. They have read and approved the final draft to be published and agreed to be accountable for all aspects of the work. Dr. David A. Solá-Del Valle is the corresponding author, who has full access to all the data in the study and takes responsibility for the integrity of the data, the accuracy of the data analysis, and the decision to submit for publication.

## Data Availability

No data are associated with this article.

## Reporting guidelines

Figshare. CARE checklist. DOI: https://doi.org/10.6084/m9.figshare.21482340.v1
