# Supplementary material for: Case Report: Reversal and subsequent return of optic disc cupping in a myocilin (MYOC) gene-associated severe Juvenile Open-Angle Glaucoma (JOAG) patient
Source: F1000Res. 2022 Nov 22;11:1361. [Version 1] doi: 10.12688/f1000research.127871.1 (PMC11167334; doi:10.12688/f1000research.127871.1)
Supplement: Peer review history

# Reviewer report: Reviewer response for version 1

**DOI:** 10.5256/f1000research.140417.r277956

**Published:** 2024-06-11

**Authors:** Nader Bayoumi

**Affiliations:** 1 Alexandria University, Alexandria, Alexandria Governorate, Egypt

The manuscript presents a case of MYOC-associated JOAG managed by a combination of surgical and medical treatments and demonstrated optic nerve cupping reversibility with treatment. The manuscript is generally well written and highlights the importance of genetic testing in cases of JOAG and childhood glaucoma in general. Specific comments for improvement of the manuscript include: 

 "To the best of our knowledge, this is the first time this phenomenon has been described in a MYOC-associated JOAG patient." Reversibility of ON cupping is reported following successful IOP control in JOAG. However, genetic analysis of JOAG patients is not routinely performed. This may account for the relative paucity of published reports about MYOC associated JOAG. Hence, please rephrase to reflect this issue; the need for routine genetic analysis of JOAG patients. 

 "the patient underwent an urgent trabeculectomy with mitomycin-C" Please explain why an angle procedure was not performed given the reported high success rate of angle procedures (in isolation or combined to filtering surgery) in JOAG.

 Table 1: Please present information on the bleb appearance over time (given the fluctuation of IOP).

 Please comment on the health of the neural rim (colour, pallor, etc) throughout the follow up period, before, during and after the reported reversibility of cupping.

 Was a VF performed at the time when the cup reversibility was noted? In other words, was the reversibility of cupping noted clinically correlated with the function of the ON? The presented VFs demonstrate improvement of PSD & MD between 2018 & 2019 visits (although the VFI deteriorated).

 "In May 2019, the patient’s IOP OS spiked to 23 mmHg and her cup-to-disc ratio increased to 0.70, indicating a return to cupping of the nerve." The patient was subsequently prescribed Timolol and the IOP control was regained. Did the patient demonstrate repeated reversal of the ON cupping with subsequent reduction of IOP? Please explain (or at least hypothesise) why not.

Are enough details provided of any physical examination and diagnostic tests, treatment given and outcomes?

Yes

Is the case presented with sufficient detail to be useful for other practitioners?

Yes

Is sufficient discussion included of the importance of the findings and their relevance to future understanding of disease processes, diagnosis or treatment?

Yes

Is the background of the case’s history and progression described in sufficient detail?

Partly

Reviewer Expertise:

Glaucoma, childhood glaucoma

I confirm that I have read this submission and believe that I have an appropriate level of expertise to confirm that it is of an acceptable scientific standard.

# Reviewer report: Reviewer response for version 1

**DOI:** 10.5256/f1000research.140417.r209578

**Published:** 2023-11-03

**Authors:** Bryan Ang

**Affiliations:** 1 Tan Tock Seng Hospital, Singapore, Singapore

Overall, this is a well-written manuscript describing an interesting case that deserves sharing with the wider scientific community. The authors should also be congratulated for successfully managing this difficult case, which has done well despite what appears to be a stormy immediate postoperative course.

 I have a few queries/comments regarding this case, and some points for the authors' consideration.

1. Pg 3 - “This is the first time this phenomenon has been described in a MYOC-associated JOAG patient.” A suggestion to the authors is to consider a more nuanced statement. This phenomenon has indeed been described in JOAG patients. While previous studies may not have definitively elucidated the MYOC mutation, it may be well plausible many were MYOC-related JOAG cases - particularly given the high overall prevalence of MYOC mutation among JOAG eyes.
2. The reliability indices for the various tests seem to have been omitted. This case report relies heavily on investigation and imaging findings. It may be useful to include these indices for better contextualization of the results – e.g. indication of the signal strength for OCT scans, and the reliability indices for HVFs.
3. Did any of the patient's siblings with the MYOC variant have a similar clinical picture? Did any of them undergo surgery with reversal of cupping as well? If this is known, it will be of interest and should be mentioned in this case report as well.
4. Pg 11 - “In the current case, we did not observe any functional changes on HVF or OCT that would correspond to the structural changes observed at the optic nerve head. Horani et al. and Tan et al. quantified the test-retest variability of HVF and OCT imaging, reporting mean variability among repeat tests of 2.44 dB on HVF and 4.89 μm on Cirrus OCT.” Authors can consider explaining the rationale for mentioning the test-retest variability of HVF and OCT imaging. Are authors suggesting that the apparent absence of corresponding changes in the HVF and OCT versus those in the degree of cupping/neuroretinal rim thickness may be confounded by the test-retest variability? While this may be conceivable for the HVFs, the direction of change in OCT findings (which does not correspond with the change in cupping/neuroretinal rim thickness) appears to be well out of the test-retest variability range.
5. Repeatability of tests – as a related point, were the tests repeated to confirm the readings/measurements? The postoperative OCTs and HVFs are likely representative and reproducible, given the consistent longitudinal readings over several visits. But how about preoperative investigations? Only one set of preoperative results appears to be presented here. It may be useful to include reliability indices (as per my previous comment (2) ) or include a mention that the results were similar for repeated tests.
6. Pg 12 - “We hypothesize that the higher preoperative RNFL value in this case may have been artificially elevated in the setting of extremely high IOP (46 mmHg)”. Why should this be? Kindly explain this association.
7. Pg 12 - “Notably, adult cases of cupping reversal are limited to patients in the early stages of glaucoma, unlike the current severe stage patient”. It appears that Esfandiari *et al*’s paper (reference 22 in the manuscript) may also have described cupping reversal in patients with advanced disease.
8. What was the preoperative duration of symptoms/duration of high IOP preoperatively for the left eye? Could this also be a factor that may influence the “reversibility” of cupping/neuroretinal rim thickness before and after surgical intervention?
9. Throughout the manuscript, OCT changes/findings have been repeatedly described as being “functional” in nature. However, OCT changes may perhaps be considered more structural in nature.
10. Was the axial length of the eye measured for this case? Axial length is correlated with RNFL thickness, as well as with IOP. If known/measured, it would be of interest and useful to mention in this case report.
11. The inconsistency/non-correspondence in findings between cupping/neuroretinal rim thickness/area versus RNFL thickness appears to be a key point of interest in this case report and may deserve further elaboration/explanation.

Are enough details provided of any physical examination and diagnostic tests, treatment given and outcomes?

Partly

Is the case presented with sufficient detail to be useful for other practitioners?

Yes

Is sufficient discussion included of the importance of the findings and their relevance to future understanding of disease processes, diagnosis or treatment?

Yes

Is the background of the case’s history and progression described in sufficient detail?

Yes

Reviewer Expertise:

Glaucoma, Glaucoma Progression, Glaucoma Surgery

I confirm that I have read this submission and believe that I have an appropriate level of expertise to confirm that it is of an acceptable scientific standard.
